# A new roadmap for social medicine curriculum design based on mixed methods student and faculty evaluations of the preclinical curriculum

**DOI:** 10.1186/s12909-021-02885-4

**Published:** 2021-08-20

**Authors:** Sheridan M. Finnie, Richard J. Brach, Christina A. Dawson, Samuel B. Epstein, Raghav K. Goyal, Karen M. Lounsbury, Shaden T. Eldakar-Hein, Timothy Lahey

**Affiliations:** 1 From the Class of 2022, Larner College of Medicine, University of Vermont, Given Medical Bldg, E-126, 89 Beaumont Ave, Burlington, VT 05405 USA; 2 Class of 2021, Larner College of Medicine, University of Vermont, Given Medical Bldg, E-126, 89 Beaumont Ave, Burlington, VT 05405 USA; 3 The Office of Medical Student Education, Larner College of Medicine, University of Vermont, Given Medical Bldg, E-126, 89 Beaumont Ave, Burlington, VT 05405 USA; 4 Department of Medicine/Ethics, Larner College of Medicine, University of Vermont, Given Medical Bldg, E-126, 89 Beaumont Ave, Burlington, VT 05405 USA

**Keywords:** Social justice, Social medicine, Racism, Health inequities, Social determinants of health, Curriculum design, Medical education, Health care

## Abstract

**Background:**

To support the development of social medicine curricula that empower medical school graduates to redress health inequities, we conducted a mixed methods student and faculty evaluation of an expanded and innovative preclinical social medicine curriculum.

**Methods:**

We implemented a longitudinal, interactive preclinical social medicine curriculum that was closely integrated with foundational science teaching then conducted a survey-based mixed methods student and faculty curriculum evaluation. Based on these results, we propose a novel conceptual roadmap for social medicine curriculum design.

**Results:**

Student and faculty evaluations of an expanded and innovative longitudinal preclinical social medicine curriculum were strongly favorable. Both student and faculty respondents indicated a particular desire for deeper coverage of race and poverty among other social medicine domains. Qualitative student evaluations highlighted the importance of faculty champions to social medicine teaching as well as the educational impact of stories that exemplify the practical impact of the social determinants of health on specific patient experiences. Qualitative faculty evaluations pointed to the challenges of curriculum integration and the need for faculty career development in social medicine teaching.

**Conclusions:**

Based on mixed methods student and faculty curriculum evaluation data, we propose a novel conceptual roadmap for the design of social medicine curricula at other institutions.

**Supplementary Information:**

The online version contains supplementary material available at 10.1186/s12909-021-02885-4.

## Introduction

Health outcomes are strongly affected not only by biomedical processes such as genetics and environmental exposures but also by powerful social factors like gender, race, poverty, and education [1, 2]. Traditionally, undergraduate medical education has focused myopically on biomedical factors to the exclusion of the social determinants of health [3]. This has inevitably led to the perpetuation of structural racism, transphobia, and other forms of structural violence, stressing the need for social justice in medicine [4, 5].

More inclusive models of human disease that incorporate the social determinants of health, such as Engel’s biopsychosocial model [6, 7], are increasingly accepted and promulgated in medical education [8]. Building on the work of Kasper et al. and Hixon et al., we refer to this more inclusive curricular models as social medicine, i.e. the “systematic study of the relationships between society, disease, and medicine.” [3, 8, 9] Social medicine centers on the principle of health care as a basic human right, as issued by the World Health Organization’s declaration of Alma-Ata in 1978, and is aligned with the Social Medicine Consortium’s definition of social medicine that asserts the need for training medical students to locate underlying causes of health and disease in conceptualizing the practice of high-quality, equitable patient care within the social reality of today [8, 10–13].

To redress the historical insufficiency of social medicine teaching, the Liaison Committee on Medical Education (LCME), the Association of American Medical Colleges and other educational leaders have promoted the incorporation of principles of social medicine into medical education [14–18].

In response, many medical schools across the country are working to incorporate social medicine into the core undergraduate medical education curriculum [3, 16, 19]. These efforts largely fall into one of three curricular models: discrete elective courses (for example SocMed in Minneapolis) [16], semester-long, required first year courses in social medicine (for example Introduction to Social Medicine and Global Health course at Harvard Medical School) [3], and fully integrated social determinants of health curricula (for example the Health Equity and Social Justice at Rutgers New Jersey Medical School) [19]. Many institutions have developed extracurricular programming that brings a select cohort of medical students in longitudinal service and advocacy work in the community such as at the Icahn School of Medicine at Mount Sinai [20].

Despite this outpouring of innovation in social medicine curriculum design, no standard model for a social medicine curriculum has emerged. One obstacle to progress may be that each institution has a unique baseline approach to social medicine teaching and thus a different pathway toward design of a fully realized social medicine curriculum. To provide a conceptual framework for all institutions conducting social medicine curriculum design, we conducted a mixed methods evaluation of an expanded and innovative preclinical social medicine curriculum at the University of Vermont’s Larner College of Medicine (UVM Larner) as recently described in more detail in this journal [21]. Based on this mixed methods curriculum evaluation, we propose a conceptual framework for social medicine curriculum design. The urgency of this work is only highlighted by the explosive COVID-19 pandemic and the manifold ways it exposed the powerful adverse effects of the social determinants of health.

## Methods

### Curriculum design

**Fig. 1:**
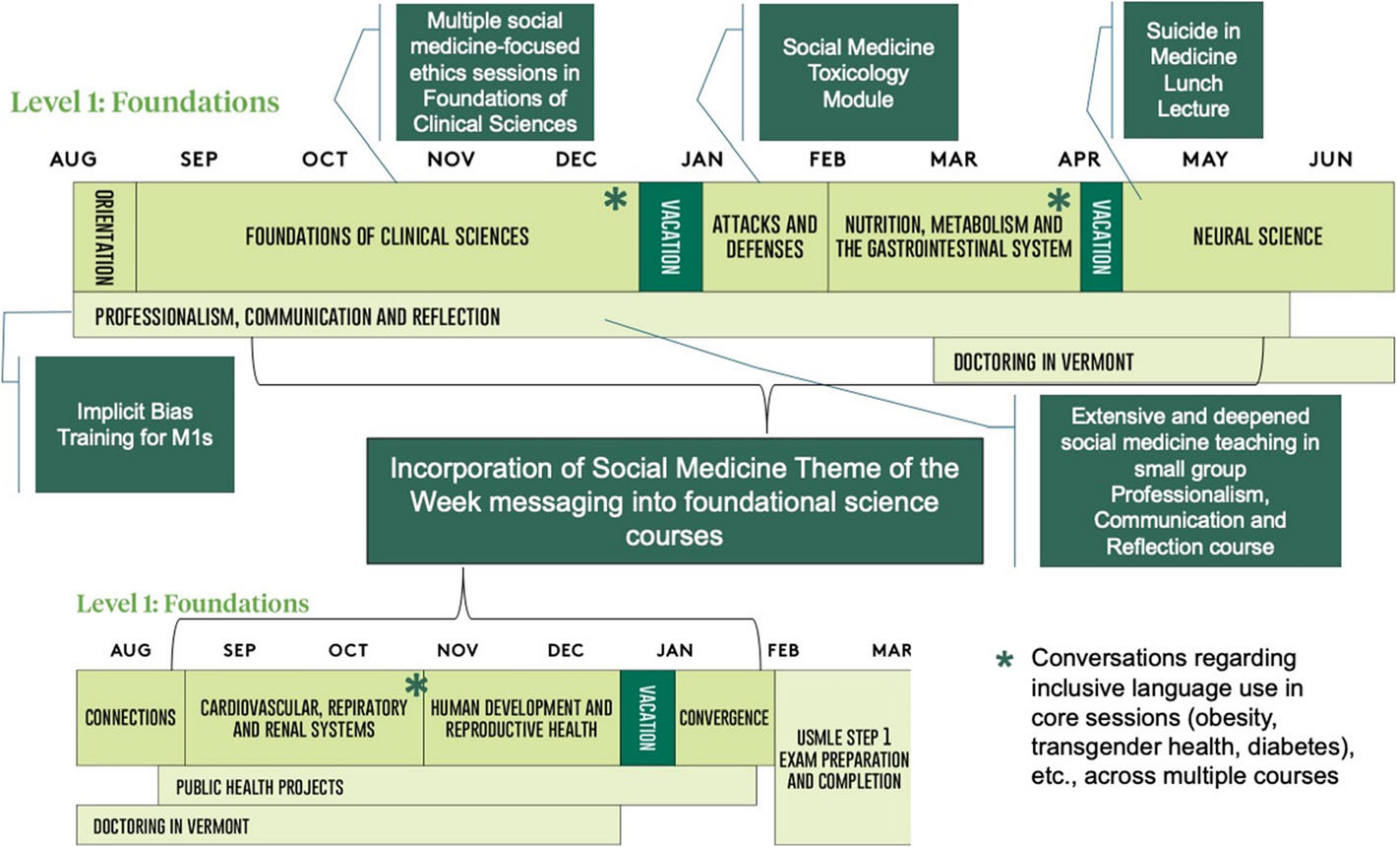
Selected social medicine additions/revisions to preclinical curriculum at UVM’s Larner College of Medicine

The social medicine curriculum at UVM Larner is required of all students and consists of three core components, largely focused in the preclinical years: (1) a strong series of related conversations regarding social medicine topics delivered in the weekly first year, small-group longitudinal discussion course with a faculty preceptor (Professionalism, Communication and Reflection course, or PCR); (2) social medicine content embedded in foundational science courses such as 15 ethics sessions in the 5-month Foundations of Clinical Sciences course; and [3] cross-curricular integration of social medicine content via student-faculty collaborations in multiple courses as well as the Social Medicine Theme of the Week (SMTW) which integrates social medicine content across the curriculum and encourages critical reflection (Fig. 1).

The SMTW provides the mechanism for cross-curricular integration through a weekly social medicine theme (ex. “Social Determinants of Mental Health” and “Aging and Bias”) that links foundational science content and PCR content with formal objectives in social medicine while promoting critical reflection. This approach involves a weekly student announcement describing that week’s theme, listing of the theme and relevant objectives in the student online scheduling portal, SMTW topic synthesis with PCR session and faculty integration of theme content or personal reflection in foundational science active learning sessions. The curricular design and implementation are described in more detail separately [21].

### Survey of student and faculty experiences of the social medicine curriculum

The student survey was emailed in 2019 to all first-year medical students in the weekly UVM Larner newsletter at the completion of the same preclinical curriculum described previously [21]. Additional emails and announcements were sent and made to the first-year class for 3 weeks following initial administration encouraging survey participation. The faculty survey was emailed to all UVM Larner faculty who teach in the first-year curriculum by the director of pre-clinical coursework. Subsequent reminder emails were sent at one- and two-week intervals.

### Student and faculty survey design

The student survey consisted of 8 multiple choice required questions as well as 3 open-ended comment questions. The faculty survey consisted of 18 required questions and 3 open-ended questions. Each faculty survey was coded with skip logic such that participants were asked to answer from 1 to 12 additional questions based on responses to prior questions. Example Likert scale questions from student and faculty surveys included, “How would you describe your awareness of the Social Medicine Theme of the Week, “How would you rate the balance of content about the following Social Determinants of Health in your first year of medical school/the Foundations medical curriculum” (student probe/faculty probe, respectively), and “Please give a specific example of a time when the Social Determinants of health were taught well in the first year curriculum.”

### Curriculum evaluation analyses

We conducted quantitative survey analyses using basic descriptive statistics including count, frequency, and percentage using STATA version 15.1 (Stata Corp., College Station, TX, USA). We analyzed textual responses employing techniques borrowed from grounded theory [22]. Student and faculty open-ended responses were coded separately using the qualitative software package Dedoose, and codes were refined throughout the coding process to develop a codebook. The lead coder (author SF) conducted line-by-line open-coding to identify emerging themes, generating frequent memos to document different analytic reflections and assist in thematic development. Coders 2 (author RB) and 3 (author TL) conducted paragraph level open-coding. Any coding inconsistencies were discussed and resolved by coders. A final codebook was iteratively refined and finalized. The finalized codebook was used to re-code a selection of the data for calibration and consistency. Twenty-one student and 13 faculty codes were identified in the text responses and used to conceptualize higher-order conceptual themes from the descriptive codes for each student and faculty data. We grouped themes into three core categories and a data map for each student and faculty data was developed to visualize the results from this generative process. We created a conceptual model for developing and delivering a social medicine curriculum at a US medical school based on the key findings of this survey and associated medical education literature.

## Results

Seventy one of 118 first year medical students (60.2%) and 40 of 178 surveyed preclinical curriculum faculty members (22.5%) responded to the surveys.

### Student curriculum evaluation results – quantitative

**Fig. 2:**
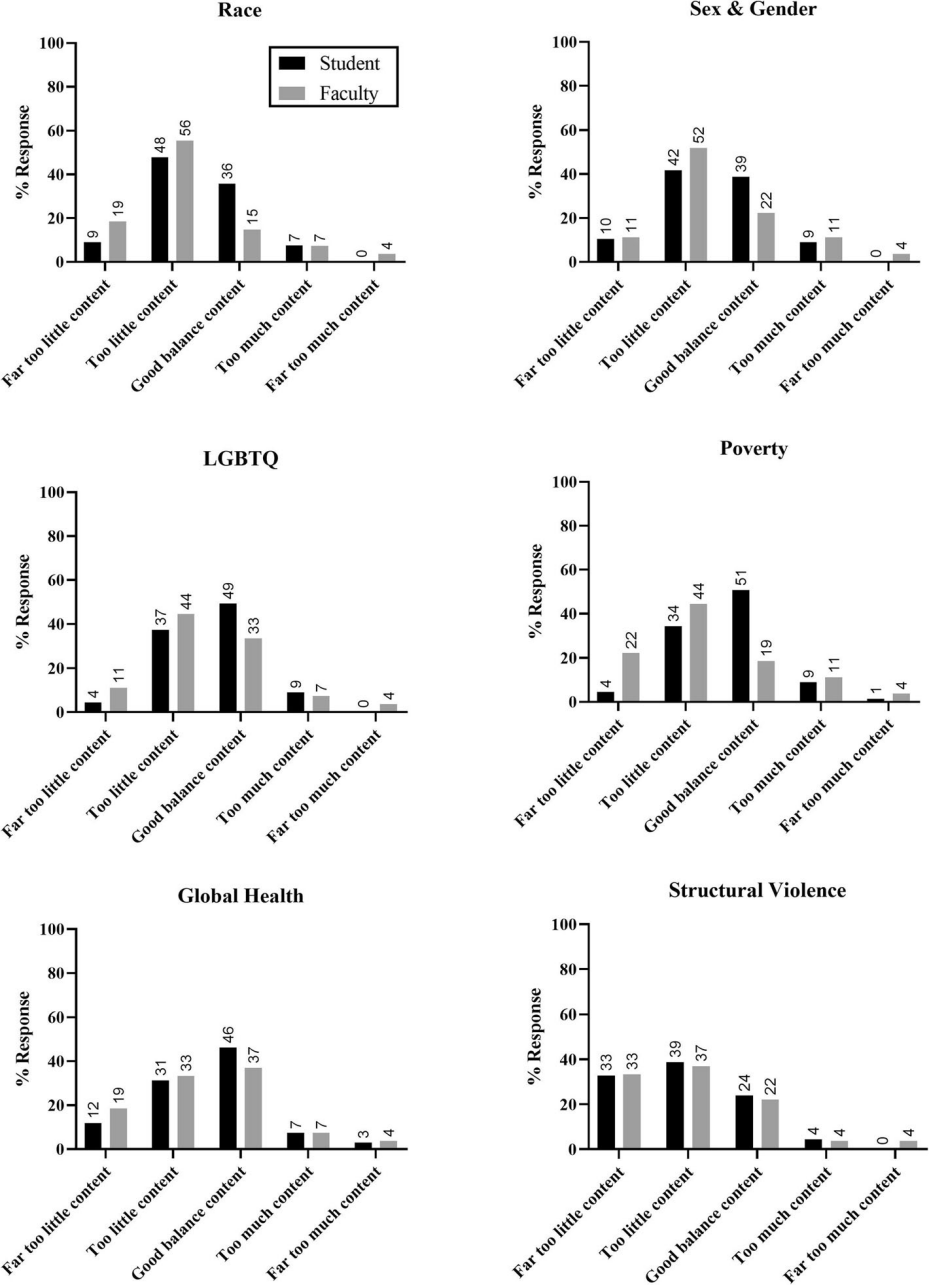
Comparison of student and faculty rating of coverage of social medicine topics across curriculum. Sample size same for all panels: student (black) *N* = 67; faculty (grey) *N* = 40

Quantitative student curriculum evaluation findings are summarized in Fig. 2.

The vast majority of student respondents were aware of the social medicine curriculum (64 of 71 respondents, 90.1%) and found the curriculum “a little helpful” or “very helpful” (56 of 62 respondents who evaluated curriculum helpfulness, 90.3%). More detailed results are summarized in Fig. 2. While most students indicated “good balance” or “too much” depth of coverage in content related to poverty, lesbian, gay, bisexual transgender and queer (LGBTQ+) content, and global health, most students reported the perception of “too little” or “far too little” content on various social medicine topics, such as race, structural violence, and sex & gender.

### Student curriculum evaluation results – qualitative

**Fig. 3:**
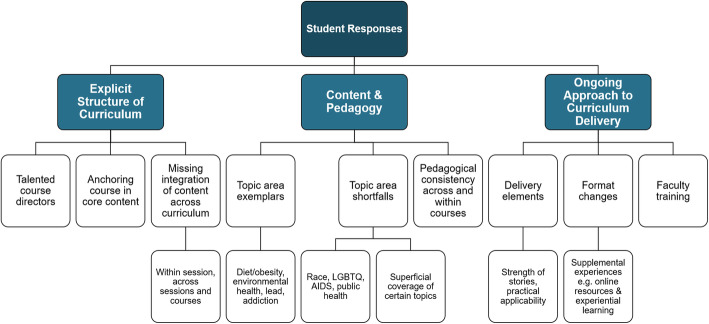
Findings of qualitative analysis of student survey responses. Boxes from bottom-up represent sequentially higher-level conceptual categories derived from the qualitative coding and category identification process

Themes from the qualitative faculty curriculum evaluation are depicted in Fig. 3.

Key findings included student desire for improved integration of content across the curriculum and cited various examples of successful implementation of social determinants of health content into the curriculum. The importance of an explicit structure of the curriculum was well illustrated in one student’s comment:

“I think introducing a framework early-on which provides some organization of different social determinants of health, and which can be revisited over time, would help provide a larger context for these discussions.”

Content and pedagogy included references to social determinants of health content integration successes that can serve as models for further integration and improvement:

“I really loved the session we had on lead poisoning and Flint, Michigan. It was the perfect balance of introducing the topic with a theme, integrating that topic into the mainstream curriculum with exam-style questions, and discussing the implications of the systemic issues within that community.”

“Please work on the HIV/AIDs [sic] curriculum. We barely scratched the surface.”

“Many instructors themselves are uncomfortable talking about this info and/or do not know how. If we had someone that was able to provide some guidance to instructors or course directors this would help.”

As for the ongoing approach to curriculum delivery, students discussed their desire for innovative curricular delivery elements, such as narrative links to social medicine topics, and supplemental experiences outside of the classroom such as experiential learning opportunities. Additionally, the need for faculty training was emphasized as an essential component of a successful curricular model. Many students cited their hope to see an expansion of the existing curriculum, integrating various noted innovations:

“I would LOVE if for every workshop, there is at least one question (think/pair/share style) where we encourage students to think how social determinants of health and the social medicine theme of the week is.”

### Faculty curriculum evaluation results – quantitative

Quantitative faculty curriculum evaluation findings are summarized in Fig. 2, above**.** About half of the faculty respondents were aware of the social medicine curriculum (21 of 40, 53%), and within the subset of faculty respondents that incorporated social medicine content into their session, all found the curriculum to be “a little helpful” or “very helpful” (8 of 8, 100%). More detailed responses from faculty respondents regarding depth of curricular coverage are summarized in Fig. 2. While many faculty respondents reported satisfaction with the depth of coverage in global health, the majority were in agreement with students regarding paucity of coverage in other topics, reporting “too little” or “far too little” content on race, structural violence, sex and gender, LGBTQ+ content, and poverty. Within the subset of faculty respondents that incorporated social medicine content, half found it “a little hard” to incorporate the content into sessions (4 of 8, 50%). Faculty respondents described barriers to incorporating social medicine content into session materials including insufficient training, worry of saying something offensive, insufficient class time, not knowing about the theme of the week, not fitting with the class content, and not thinking social medicine content is important.

### Faculty curriculum evaluation results – qualitative

**Fig. 4:**
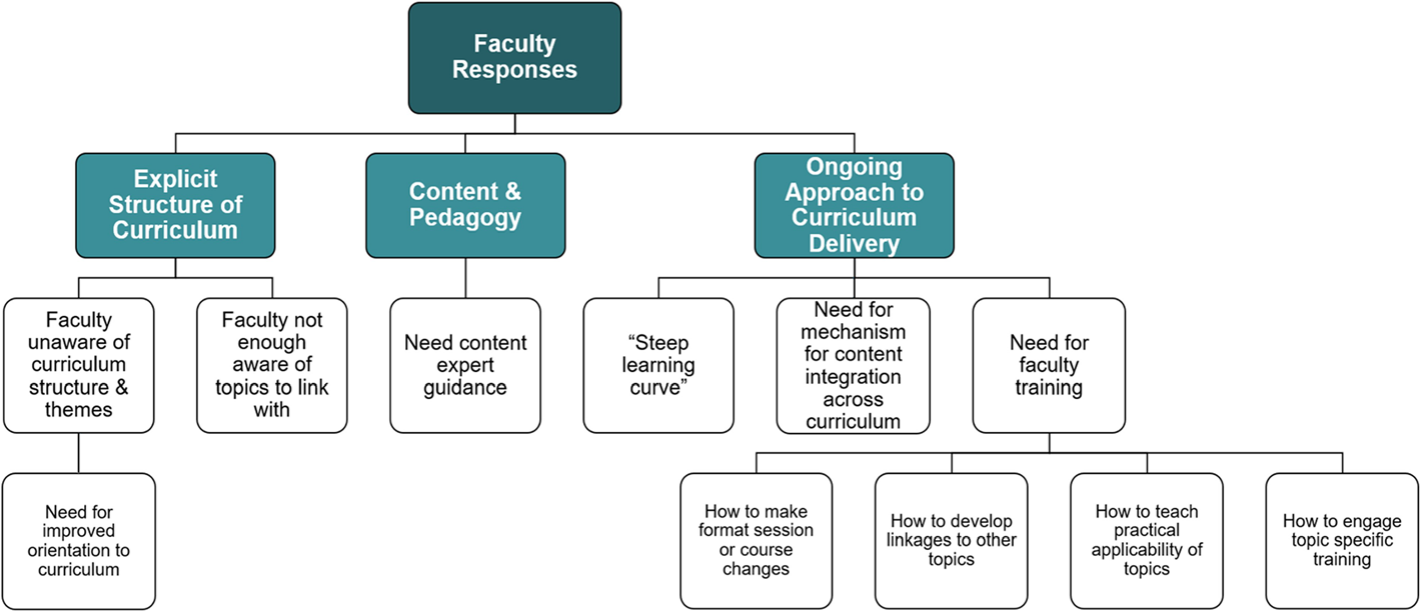
Findings of qualitative analysis of faculty survey responses. Boxes from bottom-up represent sequentially higher-level conceptual categories derived from the qualitative coding and category identification process

Themes from the qualitative faculty curriculum evaluation are depicted in Fig. 4.

Faculty respondents emphasized processes that would enhance cross-curricular integration, including increasing faculty awareness of the overarching curriculum context for their specific teaching session(s) and the need for expert guidance as well as a formal curricular leadership role.

“The provision of medical care for the poor is a core physician activity. Most physicians do it every single day. Many of them feel ill-prepared to do that work. Most physicians, by contrast, do not use knowledge of, for instance, heritable metabolic defects, on a regular basis. The mismatch between what we do teach and what we should teach makes our adult learners ask, incredulously, ‘why am I learning this stuff and not the important concepts?’”

“They [social determinants of health] should be fully integrated into what we teach as they influence so much of how patients seek and receive care, our biases and outcomes.”

“It should be seamlessly integrated into all sections with more content in those sections that are most relevant but something everywhere.”

The role of an explicit curricular structure and the need for further faculty-facing coordination regarding how to provide opportunities for students to engage with the praxis of social medicine was noted as a next step:

“We invest in teaching of biochemistry but we do not invest in teaching equitable care to marginalized populations. This results in a lack of coordination of this critical topic. Good people care about it and do it and sometimes teach about it, but they aren’t mutually aware and they don’t have a mandate to teach complementary topics so what they do is higgledy-piggeldy and our students know it.”

Despite consistent agreement with the concept of integration, challenges in reaching universal faculty awareness of the social medicine curriculum exist:

“How am I supposed to incorporate the theme into my lecture if I am unaware of the week's theme?”

As for an ongoing approach to curriculum delivery, faculty respondents emphasized the need for a mechanism to integrate the social medicine content across the curriculum. They perceived a “steep learning curve” to individual and collective implementation. To address this challenge, faculty respondents recognized the value of comprehensive faculty training in building social medicine themes into existing sessions, developing linkages of social medicine content to other topics, and emphasizing the practical nature of learning such material.

## Discussion

**Fig. 5:**
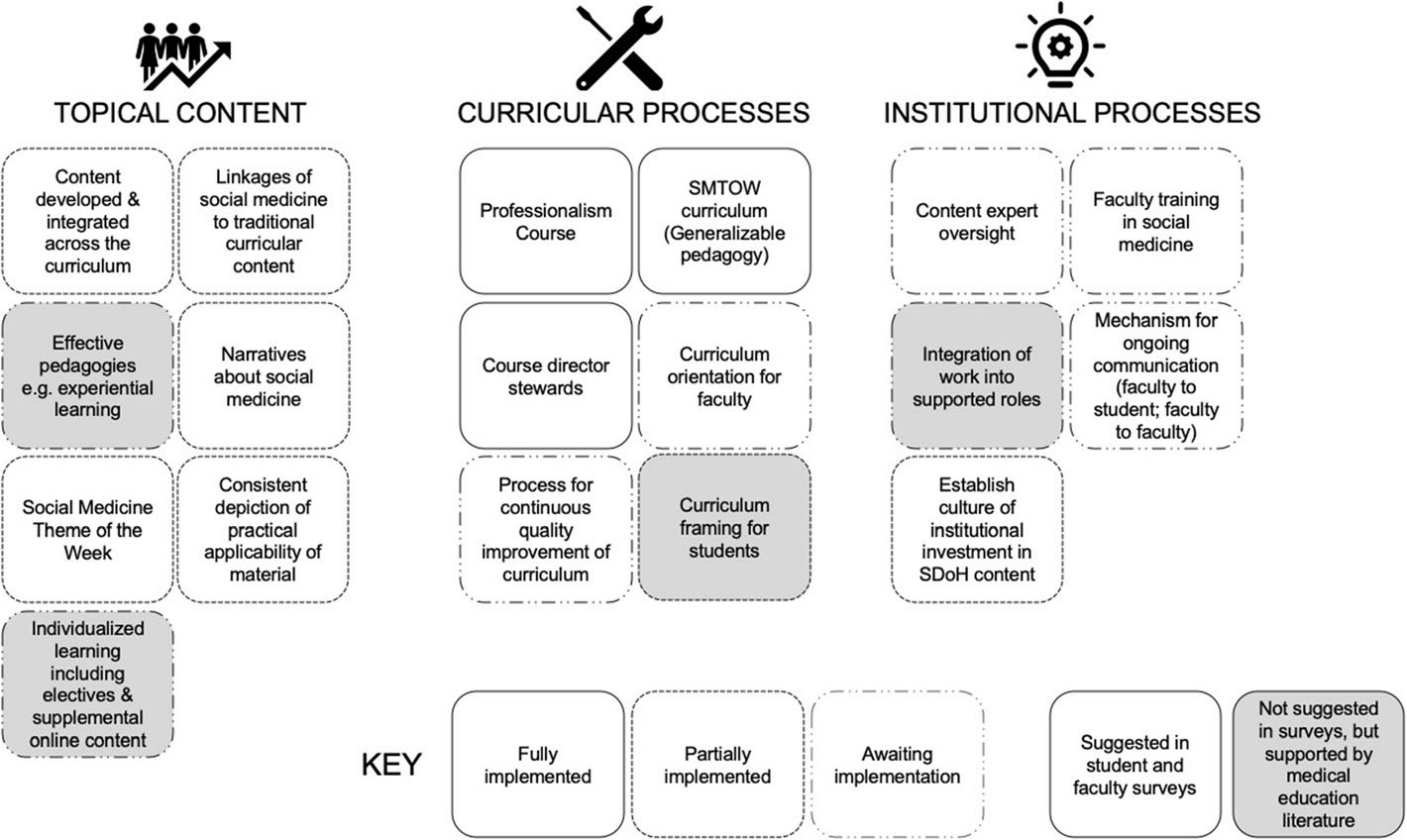
Conceptual framework for developing and delivering a social medicine curriculum at a US medical school

A mixed methods evaluation of student and faculty evaluations of an expanded and innovative preclinical social medicine curriculum at the University of Vermont’s Larner College of Medicine reveal strongly positive experiences and support the development of a novel conceptual framework for the design of social medicine teaching in undergraduate medical education (Fig. 5).

This process involves three key domains of curriculum design: social medicine content, curricular, and institutional support processes.

### Social medicine topical content

A minority of LCME-accredited medical schools report the integration of social medicine in the core undergraduate medical curriculum [3]. At the same time, racial health disparities are worsening and medical students continue to hold false beliefs that Black patients have thicker skin or experience less pain than White patients [4, 23, 24]. This speaks to a widespread need to expand coverage of *topical content* relevant to social medicine in undergraduate medical education (Fig. 5).

Our data suggest that students and faculty believe social medicine content is appropriate to include in undergraduate medical education. Also, even with extensive teaching of social medicine in the required preclinical curriculum as previously described [21], there was consensus among student and faculty respondents that even more in-depth coverage would be preferable in key topics such as race, sex and gender and LGBTQ+ issues. There will always be diverse impressions of the appropriate depth of coverage of such topics but we favor identifying through continuous quality improvement processes a depth of coverage that the majority of student and faculty respondents feel is at least not insufficient.

To promote the critical self-reflection needed to address false beliefs and implicit bias at UVM Larner, we used the PCR course and the Social Medicine Theme of the Week to develop new content and integrate it directly into the traditional curriculum, incorporate narratives and community perspectives, and consistently depict the clinical applicability of the social medicine material.

Despite these successes in integration and content development, the literature supports important next steps to fully realize the necessary topical content of a comprehensive social medicine curriculum. These include linkage of social medicine topical content to opportunities in experiential learning [25], elective training for deeply interested students [16, 26], and expansion of the social medicine curriculum into clinical education [27]. A robust foundation of social medicine content must be integrated throughout the medical curriculum to meet LCME standards as well as the imperative to prepare physician learners to address racism, structural violence, and population health needs more effectively [28, 29].

### Social medicine curricular processes

An effective social medicine curriculum involves more than generating and delivering core content. It also requires *curricular processes* that enable reflective and iterative processes that ensure continuous quality improvement, maintain content relevance, and build faculty capacity (Fig. 5) [8, 28]. The student and faculty curriculum evaluation data detailed in this manuscript are a pivotal input to this continuous quality improvement process. At UVM Larner thus far, successful curricular processes have included the involvement of course directors as key stakeholders, regular communications with faculty, and intentional awareness building of the SMTW for the student body through in-person weekly announcements and online announcements incorporated into students’ digital calendars.

Collaborations with course directors were critical to the maintenance and sustainability of the social medicine curriculum. As primary stewards of the curriculum, course directors are well positioned to maintain the curriculum as well as identify topical content most amenable to social medicine integration. Having faculty and students collaborate on the curriculum has allowed for significant bilateral teaching of all parties involved: students can keep faculty up-to-date with social medicine topics that are constantly changing, and faculty can mentor students in effective pedagogical approaches to curricula development. Through these collaborative efforts and advocacy, students have gained active experience in how to foment change that is directly translatable to health care reform work.

Course directors and their collaborators cannot sustain the social medicine curriculum alone. Additional curricular processes must engage other champions among administrative leadership and beyond. To parallel the “Social Medicine Theme of the Week,” which provides students with curricular framing, we are building a centralized notification system for faculty that informs them of the theme of the week, objectives, and other pertinent information. Additional recommendations include comprehensive faculty orientation and ongoing training in social medicine content and curricular integration, especially for facilitators of PCR where most self-reflective social medicine learning occurs. To increase sustainability and effectiveness of these processes as the extent of the curriculum and number of stakeholders grow, we recommend an annual social medicine curricular audit to facilitate ongoing communication between student leaders, course directors, teaching faculty.

### Institutional processes that support social medicine teaching

Content and curricular processes are not sufficient without building comprehensive institutional processes that support the development and implementation of a social medicine curriculum [16]. We define *institutional processes* as processes for ongoing communication, integrating curricular work, and furthering the culture of valuing the teaching and practice of social medicine (Fig. 5).

At UVM Larner, beyond centralized faculty oversight of the social medicine curriculum in close collaboration with students of the Social Justice Coalition, increased institutional investment is necessary. Three key indicated improvements at UVM Larner include (1) working toward establishing a culture of valuing and investing in the teaching of social medicine including durable funding and protected time for curricular development and faculty stewardship, (2) regular solicitation of content expert guidance via invited lectureships and other forms of inter-institutional collaboration, and (3) ongoing faculty development [30].

### Framework development

While our conceptual framework was grounded in both the findings of our survey and a literature review, other models for developing social medicine curricula exist. For example, we built on the work of researchers at the University of British Columbia who described the development of social responsibility as the necessary framework for social medicine curricula design by upholding the social contract between medicine and society with focused opportunities for students to learn outside of the classroom and clinic [14]. The 2016 National Academy of Medicine report recommends a framework for teaching social determinants of health that includes three domains of education, community collaboration, and institutional alignment [31]. Our framework elucidates content and processes that make those goals pragmatically achievable.

### Limitations & future directions

We acknowledge limitations to this study. The small sample size may not capture the full spectrum of student and faculty attitudes. Faculty members who were strong proponents of the social medicine curriculum or more closely involved may have been more likely to respond to this survey. Responses were only collected during 1 year of curriculum implementation, so may not fully capture the evolving experience of the UVM Larner social medicine curriculum. Specifically, students in the clinical portion of their training were not surveyed so our data cannot speak to the sufficiency of the later years of the social medicine curriculum. Other institutions may not have UVM Larner-specific components of the social medicine curriculum evaluated here, such as our intensive investment in active learning, our PCR course or the same electronic calendar for curriculum integration communications. Yet with growing institutional investments in similar active learning modalities we anticipate lessons learned from this context will nonetheless apply, with modifications for local applicability. Additionally, our survey hasn’t yet evaluated long-term student achievement of social medicine competencies.

Future directions of the UVM Larner social medicine curriculum include the elaboration of existing content in preclinical curriculum, deepening of coverage of social medicine in the clinical years, expansion of formal assessments of student learning, and annual surveys and semi-structured interviews of student and faculty participants that can guide future revisions. We believe the development of long-term post-curricular competencies and entrustable professional activities that result from the social medicine curriculum will be a rich field for further investigation.

## Conclusions

Students and faculty were strongly supportive of an expanded and innovative social medicine curriculum and provided well-aligned suggestions for improvement. These data provide the foundation for a novel roadmap for future curriculum development at UVM Larner and around the country.

## Supplementary Information

**Additional file 1.**

## Data Availability

The datasets used and/or analysed during the current study are available from the corresponding author on reasonable request.

## Abbreviations

LCME: Liaison Committee on Medical Education
LGBTQ+: Lesbian, gay, bisexual transgender and queer
PCR: Professionalism, Communication & Reflection course
SMTW: Social Medicine Theme of the Week
UVM: University of Vermont
