# Supplementary material for: A new roadmap for social medicine curriculum design based on mixed methods student and faculty evaluations of the preclinical curriculum
Source: BMC Med Educ. 2021 Aug 20;21:442. doi: 10.1186/s12909-021-02885-4 (PMC8376629; doi:10.1186/s12909-021-02885-4)
Supplement: Supplementary file 1 — Additional file 1. [file 12909_2021_2885_MOESM1_ESM.pdf]

Supplemental Materials for

A New Roadmap for Social Medicine Curriculum Design Based on Mixed Methods Student and Faculty Curriculum Evaluations

Sheridan M. Finnie, MPH[1*], Richard J. Brach[1]*, Christina A. Dawson[2], Samuel B. Epstein[2], Raghav K. Goyal[2], Karen M. Lounsbury Ph.D[3], Shaden T. Eldakar-Hein, MD, MS[3], Timothy Lahey MD, MMSc[4]

From the Class of 2022[1], Class of 2021[2], the Office of Medical Student Education[3], and Department of Medicine/Ethics[4], Larner College of Medicine, University of Vermont, Given Medical Bldg, E-126, 89 Beaumont Ave, Burlington, VT 05405, United States of America

*Authors contributed equally

Corresponding author:
Timothy Lahey, MD, MMSc
Professor of Medicine
University of Vermont Medical Center
111 Colchester Avenue, Smith 2
Burlington, VT 05401
Tel (802) 847-4594
Fax (802) 847-9783
Email Timothy.Lahey@UVMhealth.org
Twitter @TimLaheyMD

Evaluation of a Methodology for Integrating Social Determinants of Health into the Basic Science Curriculum for Medical Students

This survey aims to evaluate the Social Determinants of Health (SDoH) curriculum to identify key areas for curricular improvement and to understand the faculty experience of teaching SDoH material.

Hello!

Thank you for participating in this brief survey. Your time is valuable; thank you for spending it on this. We appreciate your help in improving and evaluating the SDoH curriculum.

There are 16 questions in this survey.

Faculty Questions

How would you describe your awareness of the Social Medicine Theme of the Week? *

❗ Choose one of the following answers
Please choose **only one** of the following:

◯ Never heard of it

◯ Heard of it but not sure what is involved

◯ Heard of it and aware of what is involved

How did you learn about the Social Medicine Theme of the Week? (Choose all that apply) *

Only answer this question if the following conditions are met:
Answer was 'Heard of it and aware of what is involved' *or* 'Heard of it but not sure what is involved' at question '1 [FQ1]' (How would you describe your awareness of the Social Medicine Theme of the Week?)

❶ Check all that apply
Please choose **all** that apply:

- ☐ Formal email from Dr. Lounsbury
- ☐ Formal email from Course Director
- ☐ Discovered on my own
- ☐ Heard from students
- ☐ Heard from another faculty member
- ☐ Other: ____________

How would you rate the balance of content about the following Social Determinants of Health in the Foundations medical curriculum (years 1 and 2)? *

Please choose the appropriate response for each item:

	There was far too little content on this topic	There was some helpful content, but more is needed	There was a good balance of this content	There was some helpful content, but it was more than needed	There was far too much content on this topic
**Race**	◯	◯	◯	◯	◯
**Sex & gender**	◯	◯	◯	◯	◯
**LGBTQ issues**	◯	◯	◯	◯	◯
**Poverty**	◯	◯	◯	◯	◯
**Global health**	◯	◯	◯	◯	◯
**Structural violence**	◯	◯	◯	◯	◯

Describe the role you believe the Social Determinants of Health should have, if at all, in the Foundations medical curriculum (years 1 and 2). *

Please write your answer here:

How many workshops/sessions do you facilitate during the Foundations medical curriculum (years 1 and 2)? *

Choose one of the following answers

Please choose **only one** of the following:

- ◯ 0
- ◯ 1
- ◯ 2
- ◯ 3
- ◯ 4+

What Foundations Course(s) are you part of teaching?

Only answer this question if the following conditions are met:
Answer was NOT '0' at question '5 [FQ5]' (How many workshops/sessions do you facilitate during the Foundations medical curriculum (years 1 and 2)?)

❗ Check all that apply
Please choose **all** that apply:

- ☐ Foundations of Clinical Sciences (FoCS)
- ☐ Attacks and Defenses (A&D)
- ☐ Nutrition, Metabolism, Gastrointestinal Systems (NMGI)
- ☐ Neural Sciences
- ☐ Connections
- ☐ Cardiovascular, Respiratory & Renal Systems (CRR)
- ☐ Human Development & Reproductive Health (HDRH)
- ☐ Convergence
- ☐ Other: ____________

Facilitator Questions

Did you feel you were able to successfully incorporate the Social Medicine Theme of the Week into your teaching? *

Only answer this question if the following conditions are met:
Answer was 'Heard of it but not sure what is involved' *or* 'Heard of it and aware of what is involved' at question '1 [FQ1]' (How would you describe your awareness of the Social Medicine Theme of the Week?) *and* Answer was NOT '0' at question '5 [FQ5]' (How many workshops/sessions do you facilitate during the Foundations medical curriculum (years 1 and 2)?)

Please choose **only one** of the following:

- ◯ Yes
- ◯ No

What prevented you from successfully incorporating the Social Medicine Theme of the Week into your session? (Choose all that apply)

*

Only answer this question if the following conditions are met:
Answer was 'No' at question '7 [PQ1]' (Did you feel you were able to successfully incorporate the Social Medicine Theme of the Week into your teaching?) *and* Answer was NOT '0' at question '5 [FQ5]' (How many workshops/sessions do you facilitate during the Foundations medical curriculum (years 1 and 2)?)

❗ Check all that apply
Please choose **all** that apply:

- ☐ I did not know it existed
- ☐ I felt like my lecture already included this material
- ☐ I did not have enough time
- ☐ I did not have enough training
- ☐ I did not believe it was important
- ☐ I could not fit in the material without removing essential material
- ☐ Other: ____________

Are there ways you could have been better supported to incorporate the Social Medicine Theme of the Week into your session? (optional)

Only answer this question if the following conditions are met:
Answer was 'No' at question '7 [PQ1]' (Did you feel you were able to successfully incorporate the Social Medicine Theme of the Week into your teaching?) *and* Answer was NOT '0' at question '5 [FQ5]' (How many workshops/sessions do you facilitate during the Foundations medical curriculum (years 1 and 2)?)

Please write your answer here:

How helpful did you find the Social Medicine Theme of the Week in synthesizing information regarding Social Determinants of Health into coursework? *

Only answer this question if the following conditions are met:
Answer was 'Heard of it but not sure what is involved' *or* 'Heard of it and aware of what is involved' at question '1 [FQ1]' (How would you describe your awareness of the Social Medicine Theme of the Week?) *and* Answer was 'Yes' at question '7 [PQ1]' (Did you feel you were able to successfully incorporate the Social Medicine Theme of the Week into your teaching?) *and* Answer was NOT '0' at question '5 [FQ5]' (How many workshops/sessions do you facilitate during the Foundations medical curriculum (years 1 and 2)?)

❗ Choose one of the following answers
Please choose **only one** of the following:

- ◯ Not helpful at all
- ◯ A little helpful
- ◯ Very helpful

How did you incorporate teaching about Social Determinants of Health content into your session(s)?

*

Only answer this question if the following conditions are met:
Answer was 'Heard of it but not sure what is involved' *or* 'Heard of it and aware of what is involved' at question '1 [FQ1]' (How would you describe your awareness of the Social Medicine Theme of the Week?) *and* Answer was NOT '0' at question '5 [FQ5]' (How many workshops/sessions do you facilitate during the Foundations medical curriculum (years 1 and 2)?)

❗ Check all that apply
Please choose **all** that apply:

- ☐ New slides or pre-reading
- ☐ Built on existing slide or pre-reading
- ☐ Mentioned content in class but did not create new material
- ☐ Revised case presentations
- ☐ Created discussion questions
- ☐ Other: ______

How challenging did you find it to incorporate teaching about the Social Determinants of Health into your session? *

Only answer this question if the following conditions are met:
Answer was 'Yes' at question '7 [PQ1]' (Did you feel you were able to successfully incorporate the Social Medicine Theme of the Week into your teaching?) *and* Answer was NOT '0' at question '5 [FQ5]' (How many workshops/sessions do you facilitate during the Foundations medical curriculum (years 1 and 2)?)

❗ Choose one of the following answers
Please choose **only one** of the following:

- ◯ Not challenging at all
- ◯ A little challenging
- ◯ Very challenging

How much additional preparation time did it take to integrate the content into your session?

*

Only answer this question if the following conditions are met:
Answer was 'Yes' at question '7 [PQ1]' (Did you feel you were able to successfully incorporate the Social Medicine Theme of the Week into your teaching?) *and* Answer was NOT '0' at question '5 [FQ5]' (How many workshops/sessions do you facilitate during the Foundations medical curriculum (years 1 and 2)?)

❗ Choose one of the following answers
Please choose **only one** of the following:

- ◯ <30 minutes
- ◯ 30-60 minutes
- ◯ 60 minutes+

Were there any challenges that you faced in developing and delivering this content? (Choose all that apply)

*

Only answer this question if the following conditions are met:
Answer was NOT '0' at question '5 [FQ5]' (How many workshops/sessions do you facilitate during the Foundations medical curriculum (years 1 and 2)?)

❗ Check all that apply
Please choose **all** that apply:

- ☐ I did not have prior experience with this topic
- ☐ I did not feel like I had adequate training in developing or delivering this content
- ☐ I did not know where to find credible sources
- ☐ I was unable to answer students’ questions
- ☐ I did not know how to make the material fit into the academic learning objectives
- ☐ I was worried about saying something offensive
- ☐ I did not know how to mediate conflict surrounding this topic
- ☐ I did not experience any challenges
- ☐ Other: ____________

Were there any advantages in developing and delivering this content? (Choose all that apply)

*

Only answer this question if the following conditions are met:
Answer was NOT '0' at question '5 [FQ5]' (How many workshops/sessions do you facilitate during the Foundations medical curriculum (years 1 and 2)?)

❶ Check all that apply
Please choose **all** that apply:

- ☐ The students were more engaged in the material
- ☐ I feel it made the material easier to remember and understand
- ☐ I learned something new in developing this content
- ☐ It was personally fulfilling to develop the material
- ☐ I did not experience any positives
- ☐ Other: ______________________

Please describe additional resources or supports that would have helped you better integrate the Social Determinants of Health into your teaching sessions. *

Only answer this question if the following conditions are met:
Answer was 'Yes' at question '7 [PQ1]' (Did you feel you were able to successfully incorporate the Social Medicine Theme of the Week into your teaching?) *and* Answer was NOT '0' at question '5 [FQ5]' (How many workshops/sessions do you facilitate during the Foundations medical curriculum (years 1 and 2)?)

Please write your answer here:

Thank you for completing this survey. We look forward to using your responses to improve the caliber of SDoH curriculum at LCOM and better meet the needs of the student body.

07-16-2019 – 14:45

Submit your survey.
Thank you for completing this survey.
